# A Comparative Update on the Neuroendocrine Regulation of Growth Hormone in Vertebrates

**DOI:** 10.3389/fendo.2020.614981

**Published:** 2021-02-23

**Authors:** Emilio J. Vélez, Suraj Unniappan

**Affiliations:** Laboratory of Integrative Neuroendocrinology, Department of Veterinary Biomedical Sciences, University of Saskatchewan, Saskatoon, SK, Canada

**Keywords:** growth hormone, hormones, pituitary, somatotrophs cells, neuropeptides, vertebrates, cell signaling

## Abstract

Growth hormone (GH), mainly produced from the pituitary somatotrophs is a key endocrine regulator of somatic growth. GH, a pleiotropic hormone, is also involved in regulating vital processes, including nutrition, reproduction, physical activity, neuroprotection, immunity, and osmotic pressure in vertebrates. The dysregulation of the pituitary GH and hepatic insulin-like growth factors (IGFs) affects many cellular processes associated with growth promotion, including protein synthesis, cell proliferation and metabolism, leading to growth disorders. The metabolic and growth effects of GH have interesting applications in different fields, including the livestock industry and aquaculture. The latest discoveries on new regulators of pituitary GH synthesis and secretion deserve our attention. These novel regulators include the stimulators adropin, klotho, and the fibroblast growth factors, as well as the inhibitors, nucleobindin-encoded peptides (nesfatin-1 and nesfatin-1–like peptide) and irisin. This review aims for a comparative analysis of our current understanding of the endocrine regulation of GH from the pituitary of vertebrates. In addition, we will consider useful pharmacological molecules (i.e. stimulators and inhibitors of the GH signaling pathways) that are important in studying GH and somatotroph biology. The main goal of this review is to provide an overview and update on GH regulators in 2020. While an extensive review of each of the GH regulators and an in-depth analysis of specifics are beyond its scope, we have compiled information on the main endogenous and pharmacological regulators to facilitate an easy access. Overall, this review aims to serve as a resource on GH endocrinology for a beginner to intermediate level knowledge seeker on this topic.

## Introduction

**Figure 1:**
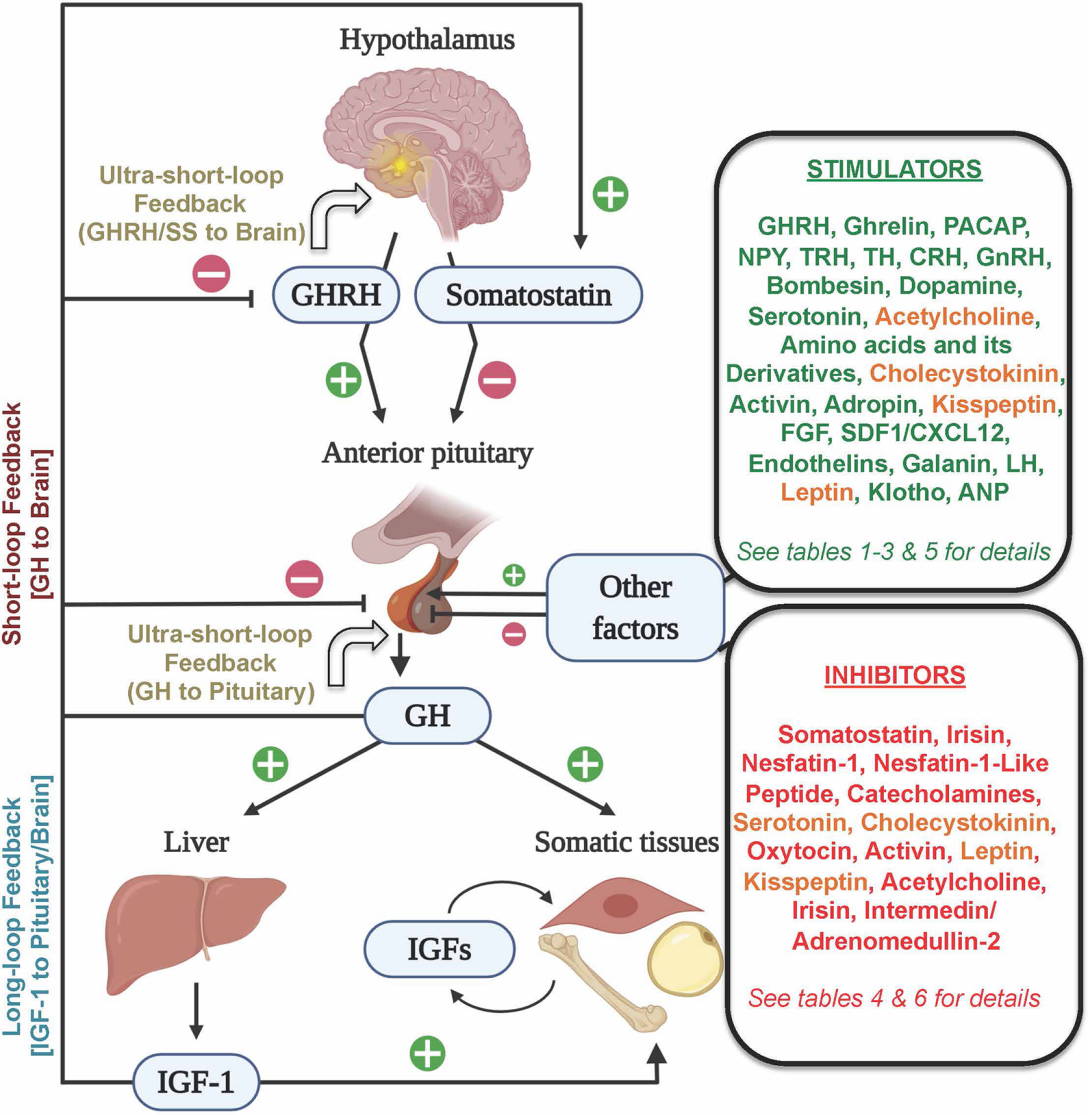
Schematic representation of the neuroendocrine (GHRH/Somatostatin–GH–IGF) axis and its main hormonal regulators. The hypothalamic stimulator GHRH and the inhibitor somatostatin mainly control GH synthesis and secretion by the pituitary somatotrophs. GH stimulates, mostly in the liver, the secretion of IGF–1, which acts in autocrine, paracrine, and endocrine manners in different somatic tissues to control diverse physiological processes, including protein synthesis, cell proliferation and metabolism. Both GH and IGF–1 could regulate its levels through the long–loop and short–loop feedback mechanisms, while GH and GHRH and somatostatin could regulate their levels via an ultra–short–loop feedback mechanism. Endogenous factors arising outside (shown in two boxes on the right side) of the hypothalamo–pituitary–liver axis could elicit stimulatory (green font), inhibitory (red font) or dual roles (orange font) to regulate pituitary GH. Figure created with BioRender.com tools.

Growth hormone (GH), originally isolated from bovine pituitaries in 1944 (1), is a key endocrine regulator of somatic growth. The main action of pituitary-derived GH is the stimulation of hepatic insulin-like growth factors (IGFs). The GH/IGF axis acts on different target tissues (**Figure 1**) including the muscle and adipose tissue, to regulate different physiological processes associated with growth promotion, protein synthesis, cell proliferation and metabolism. Therefore, dysregulation of the GH/IGF axis leads to growth disorders. In this regard, alterations in hypothalamic growth hormone-releasing hormone (GHRH), one of the main stimulators of GH (reviewed in detail below), could affect the pituitary GH and consequently the GH/IGF axis. Moreover, the disorders of the pituitary transcription factors and other components of the GH/IGF axis [GH secretagogues; GHSs, GH- and IGF-receptors, and their signal transducers] also can alter GH production, secretion and responsiveness (2).

The hypersecretion of GH, which is mostly associated with benign pituitary adenomas, causes gigantism or acromegaly (3, 4). Besides, GH excess can increase the risk of developing cancer, cardiovascular diseases, diabetes and osteopathy, and is associated with a reduction in lifespan (5, 6). The leading medical therapies for excessive GH consist of the use of somatostatin receptor ligands (SRL), as somatostatin is the main GH inhibitor (reviewed in detail below), and in the limitation of GH actions using antagonists of the GH-receptors (5, 7). However, it has been reported that some acromegaly patients become "partially resistant" to SRL treatment (8). In GH deficiency, recombinant and long-acting GH formulations are commonly used as replacement therapies for growth disorders (6, 9). Moreover, recombinant human IGF-1 replacement has been useful in reversing the adverse conditions associated with GH deficiency or GH insensitivity in children (10, 11). GH treatment was also found useful in treating some catabolic conditions such as AIDS wasting and cystic fibrosis (2, 9, 12, 13). In addition to its clinical relevance as a key molecule of the GH/IGF axis (**Figure 1**), GH is a pleiotropic hormone involved in several vital processes in vertebrates. These processes include nutrition, metabolism, reproduction, physical activity, neuroprotection, immunity, osmoregulation and even social behavior (14–21). The biological actions of GH as a major growth and metabolic modulator has been utilized in different fields including the livestock industry (22) and aquaculture (23–25). These reinforce the multidisciplinary interest on GH and the need for progress in GH knowledge across vertebrates. The identification of additional novel regulators of somatotrophs, GH synthesis and secretion has many beneficial outcomes. Some of the relatively recently identified stimulators of pituitary GH secretion or production include adropin, klotho and the fibroblast growth factors, and the inhibitors include irisin and the nucleobindin-encoded peptides nesfatin-1 and nesfatin-1–like peptide. The recent advancements in GH biology, including the regulation of GH receptors (GHR) and its signal transduction, as well as GH secretion, have been extensively reviewed in vertebrates including fish (5, 7, 14, 15, 18, 21, 26–29). The goal of this review is to serve as a one-stop resource for readers who seek beginner to intermediate level knowledge on the comparative aspects of GH endocrinology in vertebrates.

## GH Synthesis

**Figure 2:**
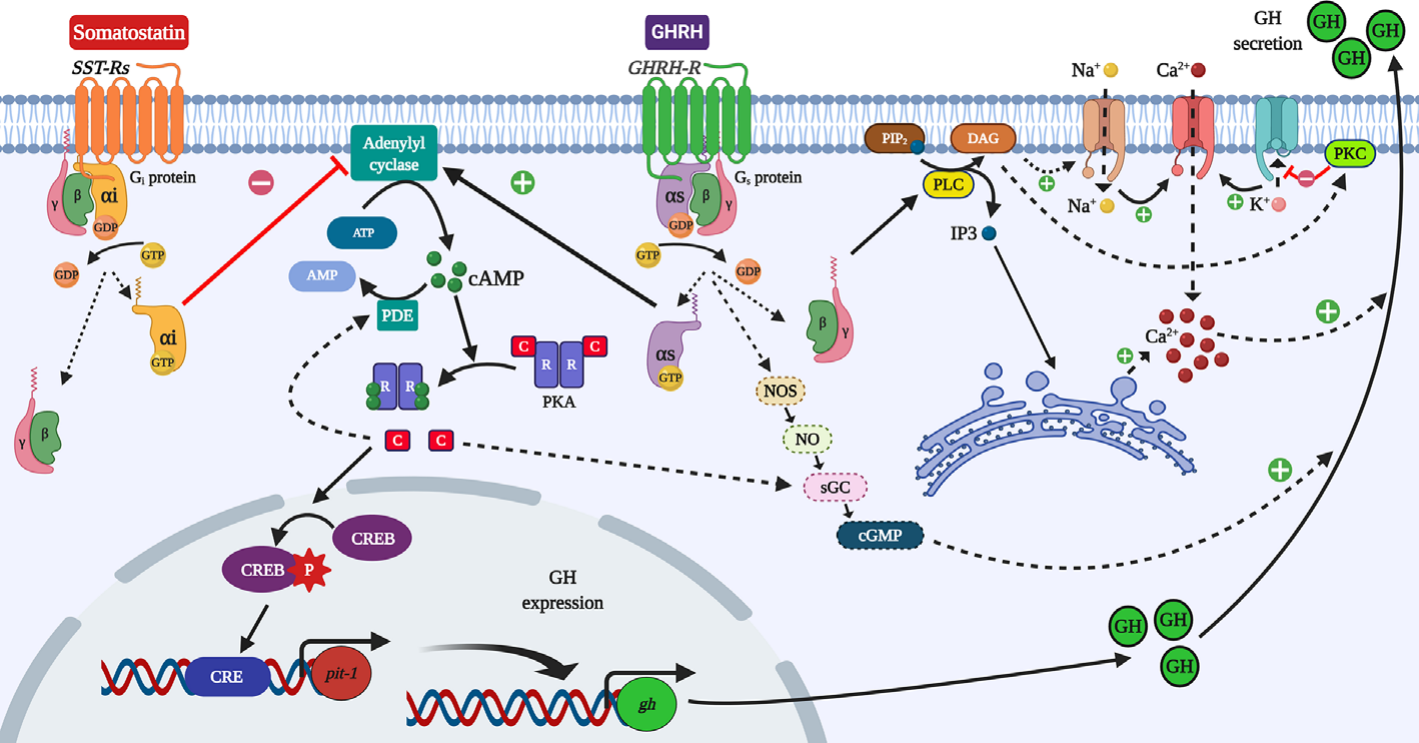
Simplified overview of the cAMP/PKA/CREB pathway in the control of GH synthesis and secretion in somatotrophs. The modulation of GH synthesis starts with the activation of GPCRs and the control of the activity of the adenylyl cyclase (AC) enzyme by the action of either stimulatory Gα (Gαs) or inhibitory (Gαi) subunits. The activation of AC increases cAMP levels, which activates the protein kinase A (PKA). CREB is one of its targets, and phosphorylated CREB can stimulate the expression of the transcription factor *pit–1*, which upregulates GH mRNAs. The stimulation of phosphodiesterases (PDE) by PKA could elicit a negative feedback, limiting cAMP levels. The activation of adenylyl cyclase and the protein lipase C (PLC) induced by GHRH causes the rise in either cAMP or IP3, respectively, stimulating the calcium (Ca^2+^) influx, which in turn potentiates the exocytosis and release of GH. This mechanism involves the activation of Na^+^ channels to depolarize the plasma membrane to regulate Ca^2+^ influx by Ca^2+^–channels, and the mobilization of Ca^2+^ from the endoplasmic reticulum. It was reported that cGMP could stimulate GH release independently of cAMP. Otherwise, the limitation of cAMP levels and the activation of K^+^ channels reduce the secretion of GH. Figure created with BioRender.com tools.

GH is mainly produced and secreted by the somatotrophs of the adenohypophysis (anterior pituitary). Generally, the modulation of these processes begins with the activation of G-protein coupled receptors (GPCRs) in the somatotrophs (**Figure 2**). The extracellular binding of GHRH to a transmembrane GPCR induces the intracellular linking of a heterotrimeric G protein (composed by α, β and γ subunits) to the GPCR (5, 30, 31). The binding of guanosine triphosphate (GTP) to the G protein induces the dissociation of the G protein and GPCR. That results also in the decoupling of Gα and Gβγ-subunits (32). In the case of a GH stimulator, the activated Gα-subunit (Gα), in turn, stimulates the adenylyl cyclase (AC) activity (33). Accordingly, the subunit involved is recognized as a stimulatory Gα (Gαs). Conversely, the binding of a GH suppressor (somatostatin) activates an inhibitory Gα-subunit (Gαi), which reduces the activity of AC (31, 33, 34). This enzyme catalyzes the conversion of adenosine triphosphate (ATP) into cyclic adenosine monophosphate (cAMP) (35). Once AC is activated, the rise of cAMP levels enables the binding of cAMP to the two regulatory subunits present in the tetrameric protein kinase A (PKA), allowing both the dissociation and activation of the two PKA-catabolic subunits (30). At this point, these activated catabolic subunits can act as serine-threonine kinases to phosphorylate a wide range of substrates, including membrane, cytosolic and nuclear proteins (30, 35, 36). Within these target substrates, the cAMP-responsive element-binding protein CREB stands out as a critical modulator of the cAMP-PKA-dependent transcriptional regulation in the somatotrophs (1, 31, 36). The phosphorylation of CREB at Ser-133 by PKA allows its binding with the cAMP response element (CRE) (30). CRE acts as a transcription factor of different cAMP-regulated genes, including the pituitary-specific positive transcription factor 1 (*pit-1*), which in turn stimulates the expression of GH gene (**Figure 2**) (15, 31, 37–39).

On the other hand, it has been reported that activated PKA can limit the levels of cAMP by either the stimulation of phosphodiesterases (PDE) (**Figure 2**), or through the desensitization of some GPCRs (36, 40–42). Besides, some GH regulators inhibit mRNA encoding their own receptors indicating that hormone desensitization also happens at the transcriptional level in somatotrophs, as observed in rats (43) and in a non–human primate (44). However, no such desensitization of a GH–inhibitory GPCR was observed in European eel (45), suggesting species–specific differences in how hypothalamic factors affect somatotrophs in mammals and teleost fish. Sex steroids regulate the fish responsiveness to both GH stimulators and inhibitors (14, 46, 47), and it is expected that the responsiveness of fish somatotrophs to hypothalamic factors follows a seasonal pattern associated with the sexual and gonadal maturation typical of each species. The distribution of pituitary endocrine cells in fish and the anatomical configuration of this gland allow the direct innervation from the hypothalamus (48, 49). This anatomical feature allows a large number of different neuroendocrine factors to be able to modulate the function of fish somatotrophs (14, 38, 50). For example, the innervation of the fish pituitary by adrenergic nerve fibers leads to the direct inhibition of GH secretion by norepinephrine in goldfish pituitary cells (51, 52). Meanwhile, in birds and mammals, norepinephrine only causes an indirect, minor effect on GH secretion that is likely dopamine–dependent (53). Thus, the regulation of somatotrophs evolved to be less complex during vertebrate evolution [reviewed by Gahete et al. (38)].

## GH Secretion

The rise in cAMP induced by the activation of AC modulates the Ca^2+^ channels to increase calcium influx, thus facilitating the exocytosis and release of GH during its stimulation (14, 35, 42, 50). During GH suppression, the negative regulation of AC blocks the Ca^2+^–channels, reducing the release of GH (31, 33). The mechanism behind these is regulated by Ca^2+^–channels. It lies in the activation of Na^+^ channels to depolarize the plasma membrane, or in the activation of K^+^ channels to hyperpolarize it, which is further regulated by the increase and decrease of cAMP, respectively (54, 55). In addition, decoupled Gβγ–subunits can modulate the protein lipase C (PLC). The activation of PLC leads to an increase in inositol triphosphate (IP3), stimulating the mobilization of Ca^2+^ from the endoplasmic reticulum (56) and enhances GH release. Furthermore, PLC in turn activates protein kinase C (PKC) (35, 44), which will also contribute to increased calcium influx by the depolarization of the membrane (**Figure 2**) (57, 58). The PLC/PKC pathway seems to be the primary intracellular modulator of some of the stimulatory actions of GHSs in mammalian and fish somatotrophs (35, 44, 59–62). During GH inhibition, in addition to the negative regulation of AC already discussed, the PLC/PKC pathway is also used to block the Ca^2+^ influx (8).

The stimulation of GH release is also mediated by cyclic GMP (cGMP) in a cAMP–independent mechanism, probably associated with nitric oxide (NO) levels (14, 50, 63). The NO/cGMP cascade could be linked to the AC/cAMP/PKA pathway as PKA can phosphorylate the soluble guanylyl cyclase (sGC) (64). Due to this, the NO/cGMP pathway also appears to be involved in the actions of GH inhibitors on somatotrophs (65). Other signaling pathways are involved in mediating the inhibition of GH secretion. Some examples include the phosphatidylinositol 3–kinase/protein kinase B (PI3K/AKT) and the mitogen–activated protein kinase (MAPK) pathways (26, 55). The different mechanisms involving cAMP, PLC/PKC, NO/cGMP, and PKA, contribute to modulate Ca^2+^ influx and the secretion of GH in vertebrates including fish. The secretion of GH in both fish and mammals follows a pulsatile, circadian pattern, with relatively higher release during the dark phase (7, 13, 14, 28, 38). Besides, sexual dimorphism in GH secretion was observed in rats (66) and humans (67, 68), with large nocturnal GH pulses and low inter–peak levels in males, and a higher interpeak and more sustained secretion in females (38). This differential GH secretion is a key determinant of the gender–specific patterns of growth and metabolism in rodents (69). GH sexual dimorphism was also reported in fish (14). This could be explained, in part, by the sex differences in hormonal regulators of somatotrophs in various species.

## Main Hormonal Regulators of Somatotrophs—GHRH and Somatostatin

Two brain (hypothalamus)–derived modulating factors, the stimulator GHRH and the inhibitor somatostatin (70), act as the primary central regulators of both synthesis and secretion of pituitary GH (**Figure 1**) (1, 7, 14, 15, 20, 21, 26, 34, 38). The hypothalamic GHRH was initially discovered from a human pancreatic tumor associated with acromegaly (71, 72). Later, GHRH was identified in non–mammals, and it was shown that fish GHRH is homologous to mammalian GHRH (73). GHRH is mainly expressed in the brain and testes in numerous vertebrates, including reptiles, birds and mammals (74), as well as in fish (73). Its main receptor is the GHRH–R (42, 75). Although it is detected in different tissues in mammals, GHRH–R is mainly expressed in pituitary cells (42). In goldfish, GHRH–R is expressed in the brain and pituitary (73). It was initially thought that GHRH–like peptides do not affect GH secretion in fish (50). Later, it was discovered that those GHRH–like peptides are indeed homologs of the mammalian PACAP (73). In the same article, the real fish homologous to mammalian GHRH was reported, and it was observed that GHRH increased both cAMP and GH secretion in goldfish pituitary cells (73). Similar GH–stimulatory effects of GHRH was identified in amphibians (76), reptiles (77, 78), birds and mammals (42, 78, 79).

Like GHRH, somatostatin is also a hypothalamic peptide, but inhibits GH secretion *in vivo* and *in vitro* in rats (80). Different forms of somatostatin, including the mammalian homolog, cortistatin, have been identified in vertebrates (38, 81). As reviewed by Sheridan and Hagemeister (26), it is now recognized that various somatostatin forms are expressed in different tissues and it is not restricted to the hypothalamus. Somatostatin exerts its effects in somatotrophs through up to five subtypes of GPCRs, the SST–Rs (26, 34, 55). The inhibitory actions of somatostatin on GH release have been widely observed in different fish (14, 26). Somatostatin blocks the transcription and translation of GH in cultured somatotrophs from rainbow trout (82), as well as blunts the stimulatory effects of other factors such as GnRH, dopamine and PACAP (14). GH in fish is under a dominant inhibitory control compared to mammals. That means, in the absence of somatostatin, the basal GH secretion reaches the maximum, and the stimulatory factors are ineffective, as observed in the turbot (83). In frogs and turtles, it was thought that somatostatin has no direct effects on somatotroph regulation, but it can block the *in vitro* stimulatory effects of TRH in amphibians and reptiles (38). Somatostatin strongly inhibits both GH mRNA and secretion from iguana pituitary *in vitro*, while the same dose and duration failed to modulate GH in chicken and rat pituitary cultures (78). In mammals, the role of somatostatin is a more complex topic. In male rats, somatostatin appears essential in generating GH secretion rhythmicity, as reviewed by Tannenbaum (84). Considering that somatostatin neurons can directly or indirectly inhibit the activity of GHRH neurons, MacGregor and Leng proposed a mathematical model to explain the hypothalamic control of GH secretion (85). However, results from other mammals, including female rats [Reviewed by Gahete et al. (38)], challenge the role of somatostatin in regulating GH rhythmicity. More recently, it has been demonstrated that somatostatin irregularly inhibits GHRH neurons in male and female mice, inducing sex–specific oscillatory patterns in the GHRH neural electrical activity (86). The sexual dimorphism in the GHRH oscillatory patterns induced by somatostatin seems dependent on the different actions of both glutamate and GABA neurons, and these differences could explain the distinctive GH secretion pattern between male and female mice (86). This topic certainly deserves further investigation in the future. In humans, it has been proposed that somatostatin regulates the magnitude of GH release but is not involved in controlling the rhythmicity of GH secretion (38, 87). In addition to their inhibitory effects, at both low and high doses, somatostatin stimulates the secretion of GH in primary porcine somatotrophs (38, 81, 88). On the other hand, SST–Rs can dimerize with other GPCRs such as ghrelin or dopamine receptors, altering the signaling of different factors and consequently, the regulation of GH (61, 89–91). Furthermore, it has been observed that somatostatin can modulate the secretion of GHRH (92), which contributes to this complex regulation. Overall, by eliciting multiple effects detailed above, somatostatin is recognized as the primary inhibitor of GH in vertebrates (7, 14, 15, 26, 38).

In addition to GHRH and somatostatin, there are several additional regulators of GH. For example, gonadal steroids can regulate GHRH effects in mice (69), contributing to the sexually dimorphic secretion of pituitary GH. Indeed, the gonadal steroids secreted during both sexual and gonadal maturation induce a clear seasonal pattern in the GH plasma levels in aquatic species (14, 93–95). Other factors including IGF–1, GH itself, ghrelin and synthetic GH secretagogs (GHSs) can modulate the synthesis and/or release of GH by somatotrophs in vertebrates (**Figure 1**) (5, 7, 15, 25, 59). The levels of IGF–1 in normal situations act as a sensor and feedback regulator of the GH/IGF system. IGF–1, which is mainly expressed in the hepatic tissue, can directly inhibit GH secretion in the somatotrophs of fish (18, 50, 96, 97), birds (98, 99) and mammals (28, 87, 92, 100), through a long–loop negative feedback (by acting on the pituitary and/or on GHRH in the brain, **Figure 1**), but also indirectly by enhancing the hypothalamic release of somatostatin (5). In addition, IGF–1 is involved in a wide range of physiological processes including protein synthesis, cell proliferation and differentiation (17, 101), and is considered the other major endocrine and local effector of the GH/IGF axis. Besides, GH itself can send feedback signals to the brain (short–loop feedback), or could act in an autocrine or paracrine manner within the pituitary (ultra–short–loop), to limit its synthesis and release by somatotrophs (**Figure 1**). Both GHRH and somatostatin are also capable of eliciting ultra–short–loop feedbacks within the brain. The exact identification of whether IGF–1 or GH induces the negative feedback in an *in vivo* model is a complex issue (38), but the inhibitory actions of GH in mammalian somatotrophs has been demonstrated both *in vitro* and *in vivo* (28). Although GH treatment increased GH in grass carp pituitary cells (50), other studies have demonstrated that GH inhibits GH release in rainbow trout pituitary *in vitro* (102). A recent *in vivo* study in gilthead sea bream showed that the administration of a sustained–release formulation of recombinant bovine GH significantly reduced pituitary GH mRNA (25). In that research, at 6 weeks post–injection, the reduction of GH mRNA was independent of circulating IGF–1 levels, supporting the negative feedback of GH in this species. To our knowledge, the ultra–short–loop has not been well characterized in the other groups of vertebrates, and future research will undoubtedly help to understand the auto–regulation of GH.

As mentioned earlier, the gradual decrease in complexity in the regulation of somatotrophs during vertebrate evolution (38) has led to a large number of factors regulating somatotrophs in fish, while a relatively shorter list of neuroregulators exists in mammals (14, 38). However, it is important to note that complexity exists in mammals. Somatostatin neurons can inhibit, directly or indirectly, the activity of GHRH neurons (103). It has been reported that GHS–receptors (GHS–Rs) can dimerize with other GPCRs, including the SST–Rs (61). The heterodimer formation (i.e. GHS–Rs:SST–Rs) could alter the signaling of the GPCRs, and thus its effects, as reported on the regulation of insulin release in rodent pancreatic cells (89). In this sense, chimeric molecules with the ability to bind with both SST–Rs and dopamine receptors induced more potent inhibition of GH release in human pituitary somatotroph adenoma cells (90, 91). Whether this enhanced potency is due to the heterodimerization of the receptors is still unknown. Additional research is needed to fully understand the implication of this mechanism in the regulation of GH secretion in somatotrophs cells along vertebrates. In addition to the dual regulation of mammalian somatotrophs (i.e. GHRH *vs.* somatostatin), a wide variety of other factors also modulate the synthesis and secretion of GH in vertebrates. The classical regulators of GH secretion in amphibians, reptiles, birds, humans and fish were reviewed by different authors in the past (14, 29, 38, 50), and more recently the knowledge on the effects of nutritional status, diet composition and environmental factors on the GH system in fish has been updated (21, 97, 104). In the present review, while revisiting the classic and main regulators of somatotrophs function in vertebrates, our focus is also on new and emerging bioactive molecules and hormones that regulate GH synthesis and/or secretion. We considered the role of these secondary GH regulators and clustered them as groups of GH–stimulatory neurotransmitters (**Table 1**), neuropeptides (**Table 2**) and peripheral factors (**Table 3**), as well as the inhibitory molecules (**Table 4**). The “up” arrows in these tables indicate a stimulatory effect, while the “down” arrows point to an inhibition. We expand on some of the major regulators below. A very detailed discussion of specifics of each of these factors is beyond the scope of this review. The readers are encouraged to consult several recent reviews of specific topics, and some are cited in this article.

**Table 1:** Summary of positive regulators of pituitary GH: Neurotransmitters.

Neurotransmitters	Groups	Roles	References
**Dopamine**	Fish	↑ GH mRNA (PKA–dependent) in tilapia	(105)
		↑ GH secretion both directly and somatostatin–dependent in goldfish	(106, 107)
		↑ GH secretion in common carp	(108)
	Amphibian, reptiles and birds	Little ↑ or no effect on GH	(109, 110)
	Mammals	↑ GH secretion in an isolated case of human acromegaly, but generally ↓ GH	(111), **Table 4**
**Serotonin**	Mammals	↑ GH secretion (somatostatin/GHRH–dependent)	(28, 38)
	Fish and birds	Opposite results also observed	**Table 4**
**Acetylcholine**	Mammals	↑ GH secretion	(112, 113–116)
	Birds	Opposite results observed in chicken	**Table 4**
**Amino acids and derivatives**	Fish	Glutamate ↑ GH secretion in rainbow trout	(117)
		Cysteamine ↑ GH secretion through somatostatin–depletion in grass carp	(118, 119)
	Mammals	Argninine ↑ GH mRNA and secretion in rat	(120, 121)
		Cysteamine ↑ GH secretion through somatostatin–depletion in sheep	(122)

**Table 2:** Summary of positive regulators of pituitary GH: Neuropeptides.

Neuropeptides	Groups	Roles	References
**Cholecystokinin (CCK)**	Fish	↑ GH secretion directly and indirectly (by reducing somatostatin) in goldfish	(123–125)
	Mammals	↑ *in vitro* GH secretion in rat	(126)
		↓ GH *in vivo* in sheep	**Table 4**
**Activin**	Fish	↑ GH release in perifused pituitary fragments of goldfish	(127)
		↓ GH mRNA in zebrafish	**Table 4**
	Mammals	↑ GH mRNA and secretion in rat, with exceptions	(128), **Table 4**
**Adropin**	Fish	↑ GH mRNA in pituitary cells of tilapia	(129)
**Kisspeptin**	Fish	↑ GH secretion in pituitary cells from goldfish	(130, 131)
	Mammals	↑ GH secretion in peripubertal rats	(132)
		↑ GH secretion in fasted sheep (ghrelin–NPY dependent), but could also ↓ it	(133), **Table 4**
**Fibroblast Growth Factor (FGFs)**	Mammals	↑ GH secretion in rat pituitaries and human adenoma cultures	(134)
**Chemokine derived factor 1 (SDF1, *aka* CXCL12)**	Mammals	↑ GH mRNA and secretion in rat	(135, 136)
**Endothelins**	Mammals	↑ GH secretion – ghrelin dependent– in bovine	(137, 138)
**Galanin**	Fish	↑ GH release *in vivo* or *in vitro* in coho salmon and goldfish	(139)
	Birds	↑ GH secretion acting directly on the pituitary	(140)
	Mammals	↑ GH release directly and indirectly	(141–145)
**LH**	Fish	Essential for GH synthesis and release in grass carp	(50, 146)

**Table 3:** Summary of positive regulators of pituitary GH: Peripheral factors/other factors.

Neuropeptides	Groups	Roles	References
**Leptin**	Fish	↓ GH mRNA	**Table 4**
	Mammals	↑ GH secretion directly and indirectly (i.e. somatostatin) in pig perifused pituitaries	(147, 148)
		↑ GH secretion in sheep	(149)
		↑ GH secretion in anterior pituitary explants of fasted bovids	(150)
		The lack of leptin receptor ↓ both GH mRNA and protein in mice	(151)
		Administration ↑ increases pituitary GH content in leptin–deficient obese mice model	(152)
**Klotho**	Mammals	↑ GH secretion in *vitro* and *in vitro* in rodents, and in human GH–secreting adenomas	(134)
**Atrial and ventricular natriuretic peptides**	Fish	↑ GH release in tilapia cultured pituitaries	(153)

**Table 4:** Summary of negative regulators of pituitary GH.

Molecules	Groups	Roles	References
**Catecholamines** [norepinephrine (NE), epinephrine and dopamine]	Fish	NE and epinephrine ↓ basal GH release from pituitary cells of goldfish	(106)
	Birds	NE ↓ GHRH–effects in chicken pituitary	(110)
	Mammals	NE ↓ basal and GHRH–stimulated GH release in cultured ovine pituitary cells	(154)
		Dopamine ↓ GH mRNA and secretion in sheep, cattle and human neonates, but opposite role also observed	(143, 155, 156), **Table 1**
**Serotonin**	Fish	↓ GH *in vitro* secretion in goldfish	(157)
	Birds	↓ GH secretion –hypothalamus–dependent– in chicken	(158)
	Mammals	Opposite role observed	**Table 1**
**CCK**	Mammals	↓ GH in sheep, but opposite role observed in rat	(159), **Table 2**
	Fish	Opposite role observed in goldfish	**Table 2**
**Oxytocin**	Mammals	↓ GH secretion in rat	(160)
**Activin**	Fish	↓ GH expression in cultured pituitaries of zebrafish	(96)
		Opposite role observed in goldfish	**Table 2**
	Mammals	↓ GH mRNA in rat, but opposite role also observed	(161), **Table 2**
**Leptin**	Fish	↓ GH mRNA in tilapia	(162)
	Mammals	Opposite role observed	**Table 3**
**Kisspeptin**	Mammals	Endogenous kisspeptin can ↓ GH secretion through GPR54 in sheep, but opposite role also observed	(163)
	Fish	Opposite role observed in goldfish	**Table 2**
**Irisin**	Fish	↓ GH mRNA and secretion in tilapia *in vitro*	(164)
**Intermedin/Adrenomedullin–2**	Mammals	↓ GHRH–stimulated GH release in rat dispersed pituitary cells	(165)
**Acetylcholine**	Birds	↓ GH secretion –hypothalamus–dependent– in chicken	(168)
	Mammals	Opposite effect observed	**Table 1**
**Nesfatin–1 and NLP**	Mammals	↓ GH mRNA and protein in rat pituitary cells	(166)

### OTHER STIMULATORS OF GH

#### Pituitary Adenylate Cyclase–Activating Polypeptide (PACAP)

PACAP was originally isolated from the ovine hypothalamus due to its AC–stimulatory effects in rat pituitary cells (167). PACAP presents two molecular forms (PACAP27 and PACAP38) (167, 168) that are expressed in the brain and other peripheral tissues (169). Initially, it was thought that in fishes and other non–mammals, both GHRH and PACAP were encoded in the same gene, whereas in mammals, they originated from different precursors (14, 170). However, later it was reported that in both non–mammals and mammals, PACAP and GHRH are encoded in different genes (169). Regarding receptors, three different subtypes (i.e. PAC1–R, VPAC1–R, and VPAC2–R) have been identified, and they can activate diverse pathways, including the signaling through AC (169). PACAP is a key GH–release stimulator, which acts through the increase of Ca^2+^ influx in fish and amphibians (15, 38, 96, 170–172). In this sense, PACAP has been postulated as the GHRH ancestor in less evolved vertebrates (50, 170, 173). Contrarily, GHRH exerts a stronger stimulation of GH release than PACAP in chicken somatotrophs (174), and the same occurs in mammals including humans (170). The role of PACAP in the secretion of GH in mammals is controversial (38, 173). It has been observed that while PACAP stimulates GH gene expression in birds, it has no such effects in rodents (78). Therefore, as previously reviewed by Gahete et al. (38), the role of GHRH and PACAP could have evolved during the evolution of vertebrates. While both GHRH and PACAP exert equipotent action regulating GH in amphibians and reptiles, in birds and mammals, PACAP only plays a secondary role, with GHRH being the main GH stimulator in those groups (170).

#### Neuropeptide Y (NPY)

NPY was first isolated from the pig brain (175) and is a member of a family of peptides that includes three (i.e. peptide Y, peptide YY, and the pancreatic polypeptide) additional GPCR agonists (176–178). NPY was identified later in different fish species (179, 180). In mammals NPY has been located in the brain (176), although NPY immunoreactivity was detected in several tissues in vertebrates, including the fish pituitary (179, 180). NPY is recognized as one of the most important regulators of energy homeostasis and food intake in both fish and mammals (178, 180). Besides, NPY acts on fish somatotrophs to increase GH secretion both *in vitro* and *in vivo* (181–183). To our knowledge, there are no published reports on the involvement of NPY on GH regulation in birds, reptiles and amphibians. The role of NPY in mammals is controversial, and appears species–specific: stimulates GH secretion in swine (184) and cows (185), in rodents NPY reduced GH (186), increased it (187), or had no effects on the secretion of GH (188). A more recent work reported that NPY stimulates the secretion of GH through its action at the hypothalamic level by the control of GHRH and somatostatin in sheep (133). Additional work is necessary to clarify whether NPY exerts a direct action on mammalian somatotrophs.

#### Thyrotropin–Releasing Hormone (TRH)

TRH, the first hypothalamic hypophysiotropic factor characterized, was initially isolated from the porcine and ovine hypothalamus in 1969 (189, 190). TRH is mainly expressed in the hypothalamus of fish, amphibians, birds and mammals, and has been detected in a number of peripheral tissues in reptiles (74). TRH binding was initially observed in the plasma membrane of the anterior pituitary extracted from cattle (191), and the receptor was later identified as a GPCR in mice (192). Although the main role of TRH is the stimulation of the synthesis and release of the thyroid stimulating hormone (TSH) to control the thyroid gland (192), TRH influences the secretion of other pituitary hormones (38). In fact, TRH increases GH expression or secretion in some fish species (14, 50), amphibians (193), reptiles (78), birds (78, 194) and mammals (78, 195). In chickens, TRH stimulates GH with a potency similar to that of GHRH (99). On the other hand, TRH could also indirectly stimulate GH through thyroid hormones (see below).

#### Thyroid Hormones (THs)

THs exist in two forms, the predominant circulating T4, and the biologically active T3 (196), which are essential components of the pituitary–thyroid axis (192). In mammals, it is well known that TRH induces the synthesis of TSH by the pituitary, which in turn induces the synthesis and release of T4 by the thyroid gland. Then, T4 can be enzymatically converted by deiodinases to T3 in different tissues, including the brain and liver (192, 196). THs can regulate the transcription of different target genes, mainly through their interaction with nuclear receptors (TRs) (196, 197). THs can also modulate gene expression through non–genomic actions involving the activation of different signaling pathways (196, 198). In fish, TRs have been found in the pituitary (50), and little evidence exists on the non–genomic actions of THs in these species [recently reviewed by Deal and Volkoff (196)]. The transcriptional regulation induced by the THs contributes to the modulation of various physiological processes, including development, growth and metabolism. In fact, THs stimulate the synthesis and release of pituitary GH in some fish (199, 200) and rats (201–204), though contrary or no effects have been observed in other fish species, reptiles or birds (15, 196). Not much is known about the TH regulation of GH in reptiles and amphibians (205), and the discrepancies in TH effects on GH regulation among fish have been recently summarized by Deal and Volkoff (196).

To our knowledge, the reasons behind the inconsistencies in the regulation of GH by THs are not fully understood. A combination of two different factors could have contributed to these contradictory results. As previously discussed by Giustina and Wehrenberg (206), the maintenance of basal GH secretion, to some extent, depends on the stimulation of somatotrophs by THs. Otherwise, when the concentration of THs exceeds the physiological level, it can increase the secretion of somatostatin and decrease GHRH, eventually causing a downregulation of pituitary GH (206). On the other hand, it has been reported that THs can stimulate the synthesis and release of hepatic IGF–1 in both fish and mammals (207–209), which through the long–loop negative feedback (**Figure 1**) could elicit a suppression of pituitary GH secretion. Based on these, the different effects of TH on GH observed in various species are likely caused by the high doses of THs used in those studies (i.e. excess vs. physiological levels), or by its effects on hepatic IGF–1. This aspect requires future confirmation through additional research.

#### Corticotropin–Releasing Hormone (CRH)

CRH, also known as corticotropin releasing factor (CRF), was first identified in ovine hypothalamus (210) and later in other vertebrates, including fish and amphibians (211). Although recognized as a hypothalamic hormone, CRH was located in other human tissues (212, 213). CRH exerts its actions through GPCRs (214). In the pituitary, CRH stimulates the secretion of adrenocorticotropic hormone (ACTH) (180, 213) and can induce the release of other pituitary hormones such as α–melanocyte–stimulating hormone (α–MSH) and β–endorphin (215). Similarly, CRH is recognized as a GH–release stimulator in non–mammals (38). While CRH stimulates GH in reptiles (77) and in European eel, but had no effects in turbot (83). CRH is a potent stimulator of TSH release from the pituitary of amphibians, fish and birds [reviewed by De Groef (216)]. Some of the CRH effects could be attributed to its indirect action via thyroid hormone stimulation. Although CRH was not considered a stimulator of GH in mammals (217), a paradoxical increase of GH in response to CRH was observed in patients with pituitary adenomas (38) and this is a topic under consideration in current research (218, 219).

#### Gonadotropin–Releasing Hormone (GnRH)

GnRH was first isolated in the early 1970s from the porcine hypothalamus (220). As recently reviewed by Duan and Allard (221), GnRH has been identified in a wide range of vertebrates, including fish, amphibians, reptiles, birds and mammals. This hypothalamic factor exerts its action in the pituitary cells through the activation of GPCRs and the signaling by PLC and cAMP pathways (222, 223). As a result, GnRH regulates the secretion of FSH and LH and is recognized as a critical modulator of the reproductive axis (224). Goldfish somatotrophs express GnRH receptors (225, 226). GnRH stimulates the secretion of GH in goldfish (227–229), tilapia (105) and Ricefield eel (230), but not in some others as found in African catfish or rainbow trout (231, 232). However, it was observed in pituitary cell culture of rainbow trout, GnRH stimulates GH secretion only in the presence of IGF–1 (233). GnRH could indirectly stimulate somatotroph function through the paracrine action of LH, which can also act as a stimulator of GH as reported in grass carp (50, 146) (**Table 2**). The regulation of GH by GnRH is a species–specific response depending on the presence of IGF–1 or other factors such as LH, or the type of receptor involved. Although it was postulated that the GH secretagogue actions of GnRH could be restricted to fish (38), recently it was found that GnRH stimulates GH secretion in the iguana, and both GH mRNA and GH secretion in chickens (78). Besides, GnRH combined with enkephalin increased GH secretion in rat pituitary cells (234), and long–term treatment with GnRH in humans caused an increase in height in precocious puberty (235).

#### Bombesin (BB)

Bombesin was first isolated from the skin of frogs in 1970 (236). BB immunoreactivity was found in reptiles (237), and two homologs, GRP and neuromedin B, were found in birds and mammals (238, 239). BB expression was also found in different fish species (240, 241), as is the case of the forebrain and pituitary of goldfish (241). Among other functions, BB stimulates the secretion of gastric acid and pancreatic enzymes (238) and is involved in the modulation of the stress response (239). As an activator of GPCRs (242), it has been reported that BB stimulates GH secretion in rat both *in vivo* (243) and *in vitro* (244). Contrarily, other authors found that BB reduced GH secretion in rats by the stimulation of somatostatin release (245). This controversial response could be associated with the presence of estrogens, as it was reported that bombesin inhibits GH secretion in normal rats but exerts stimulatory role in estrogenized rats (246). In contrast, BB stimulated GH secretion in cultured bovine pituitary cells (112). In goldfish, the perifusion of pituitary with BB significantly increased GH secretion (241). In the same species, BB increased GH release and inhibits the expression of somatostatin (123, 124), and it has been postulated that the actions of BB in the regulation of GH in these species could be mediated by somatostatin (14). However, whether BB can regulate GH synthesis and secretion in other fish, amphibians, reptiles and birds is unknown.

#### Ghrelin

Ghrelin was originally reported in 1999 (247), as the first and only known endogenous ligand of the growth hormone secretagogue receptor 1a (GHS–R1a) (248). It was purified from the stomach extracts of rats, and was later identified in a number of species from humans to invertebrates. The N–terminal region of ghrelin is very highly conserved across species, and in most species, the third serine has an octanoyl group (249). This highly conserved region with the acyl group is considered to be the bioactive core of ghrelin, and it is critical for ghrelin binding to its receptor (249). GHS–R1a, currently known as the ghrelin receptor, is expressed in the pituitary somatotrophs and allows the direct action of ghrelin on these cells to induce GH synthesis and secretion. Ghrelin is known to stimulate GH secretion in many species including rats (250, 251), humans (252, 253), birds (103, 254), and fish (255–260). These effects are either *in vitro*, supporting the ability of ghrelin to act directly on somatotropes, or *in vivo*, by acting directly or through influencing the multitude of other GH regulators (14, 50). The binding of acylated ghrelin to its receptor triggers a cascade of intracellular events, including the stimulation of phospholipase C, inositol triphosphate and calcium pathways (261, 262). Overall, almost two decades since its discovery, ghrelin is now considered as one of the most important hormonal regulators of GH in vertebrates.

#### Other Stimulators

In this review, we have tabulated the neurotransmitters (**Table 1**), neuropeptides (**Table 2**) and the peripheral factors (**Table 3**). Their specific effects in different species or groups are also furnished in these tables. In addition to the factors already discussed, a wide variety of other minor factors have been shown to exert a GH stimulatory role. For example, it has been recently reported that the peptide hormone adropin, which participates in the regulation of vascular function and energy homeostasis in mammals, stimulates GH gene expression in the pituitary of tilapia (129). To our knowledge, it is unknown whether adropin participates in the regulation of GH secretion in other groups of vertebrates. On the other hand, it has been observed that the transmembranal protein klotho, originally recognized as an ageing–suppressor in mice, increases GH secretion both *in vitro* and *in vitro* in rodents, as well as in human GH–secreting adenomas (134). Moreover, klotho is a modulator of the IGF–1 signaling pathway. It can inhibit the peripheral actions of IGF–1, and block the negative feedback of IGF–1 on pituitary GH secretion (92). Consequently, klotho has been postulated as a new player in the regulation of GH/IGF axis in mammals (92). However, the potential role of klotho in the regulation of GH secretion in other groups of vertebrates, including fish, is unknown. Besides, klotho can also regulate the signaling pathway of the fibroblast growth factor (FGF) (134). The same authors have also observed that FGF increased GH secretion in both rat pituitaries and human adenoma cultures (134). While it is unknown whether FGF exerts a direct action on GH regulation in other vertebrates, it has been recently observed that FGF increases the secretion of ghrelin in zebrafish (263). Thus, it is expected that FGF could also influence (at least indirectly) GH levels in fish. Certain amino acids, including aspartic acid, glutamic acid and arginine, although recognized as classical regulators of GH, was thought not to act directly on somatotrophs (264–266). However, new *in vitro* studies have shown that some amino acids exert their effects directly at the pituitary level (**Table 1**). However, it is important to note that some molecules may have species–specific roles and exert inhibitory actions, as detailed in **Table 4**. In addition to the endocrine regulators of GH discussed here, a large number of pharmacological compounds were employed to study the regulation of GH in somatotrophs. We have summarized the main pharmacological stimulators (**Table 5**) of the major signaling pathways involved in the regulation of both the synthesis and secretion (**Figure 2**) of GH. For further details on the use of these molecules, and the most effective doses or concentration ranges reported, please refer to the literature cited in the table.

**Table 5:** Selection of GH signaling pathway stimulators.

Target/category		Molecules	Doses	References
**G protein**	Activator of stimulatory Gα subunits (Gαs)	Cholera toxin	0.025–25 ng/mL, 3 nM	(267, 268)
	Blocker of inhibitory Gα subunits (Gαi)	Pertussis toxin	10–300 ng/mL	(135, 165)
**Adenylyl cyclase**		Forskolin	0.01–10 µM	(43, 105, 146, 173, 267, 269, 270)
**PKA**	Cell permeable cAMP analogs	8–bromo–cAMP	0.3–5 mM	(172, 268, 271)
		8–pCPT–cAMP	40–500 µM	(166, 272)
	Inhibitors of phosphodiesterases	IBMX	0.001 mM–10 µM	(267, 269, 271)
		Rolipram	10 µM	(269)
**CREB**		TUDCA	200 µM	(273)
**Calcium levels**	Ionophores	A23187	3–30 µM	(172, 268)
		Ionomycine	10 µM	(171, 274)
	Voltage–sensitive calcium channels (VSCC)	Bay K8644	10 nM–10 µM	(172, 274)
	Inhibitors of Ca^2+^–ATPase (SERCA)	Cyclopiazonic acid and BHQ	10 µM	(274)
		Thapsigargin	100 nM	(88, 262)
	Activators of Ca^2+^ release channels	Caffeine	10 mM	(274)
		Ryanodine	0.01–100 nM	(275)
**Nitric oxide route**		SNAP	0.01–1000 nM	(148)
		L–AME	1 mM	(65)
**PLC**		m–3M3FBS	10 µM	(276)
**PKC**		PMA	0.1–1 µM	(59, 83, 277)
		DiC8	10 µM	(278)
**PI3K**		sc3036	10 µM	(279)
**JAK2**		Coumermycin A1	1 µM	(280)

## Other GH Inhibitors

Other factors with an inhibitory role on GH are summarized in **Table 4**. Note that some molecules may have species–specific roles and exert the opposing actions, as detailed in **Tables 1****–****3**. For example, it has been reported that irisin, which is recognized as a metabolic peptide in mammals, inhibits both GH mRNA and secretion in cultured pituitary cells of tilapia (164). It is unknown whether irisin has a direct modulatory role on GH synthesis and secretion in other vertebrates. An inverse association between GH and irisin levels has been observed in humans, as the administration of recombinant human GH in young patients with Turner syndrome increased the circulating levels of irisin (281). We have reported that two novel metabolic peptides, nesfatin–1 and nesfatin–1–like peptide, are negative modulators of the synthesis of pituitary GH in mammals (166). Although their receptors are still unknown, it is expected that these peptides act through GPCR (282). It has been shown that both nesfatin–1 and nesfatin–1–like peptide regulate GH in the rat somatotrophs through the AC/PKA/CREB signaling pathway (166), suggesting that the mechanism of action of nesfatin–1 and nesfatin–1–like peptide involves a GPCR associated with an inhibitory Gα–subunit (Gαi). As discussed in the previous section, numerous pharmacological inhibitors were also used for the study of GH and somatotrophs **(****Figure 2****)**. These are listed in **Table 6**.

**Table 6:** Selection of GH signaling pathway inhibitors.

Target/category		Molecules	Doses	References
**G protein**	Blocker of stimulatory Gα subunits (Gαs)	Suramin, and its analogs	10 µM	(283)
	Activator of inhibitory Gα subunits (Gαi)	*Pasteurella multocida* toxin	1 nM	(284)
**Adenylyl cyclase**		MDL–12330A	0.03–30 µM	(88, 172, 262)
**PKA**	Blockers	H89	100 nM–30 µM	(105, 172, 262, 268)
		Rp–cAMP and DPT–PKI	50 µM–1 mM	(230, 268)
	Phosphodiesterase activator	MR–L2	1–10 µM	(285)
**CREB**		2–naphthol–AS–E–phosphate	25 µM	(286)
**Calcium levels**	Cell permeable Ca^2+–^chelator	BAPTA–AM	10–50 µM	(135, 171, 274)
	Voltage–sensitive calcium channels (VSCC)	Nifedipine and Verapamil	1–100 µM	(130, 230, 262, 271)
		Ca^2+^ antagonists CoCl_2_ and CdCl_2_	0.1–2 mM	(88, 271)
	Activator of Ca^2+^–ATPase (SERCA)	CDN1163	10 µM	(287)
	Inhibitors of Ca^2+^ release channels	TMB–8	100 µM	(274)
		Xestopongin C	1 µM	(275)
**Nitric oxide route**		NMMA	0.3–1 mM	(148, 268)
		NAME	10 µM	(65)
**PLC**		U–73122	5–50 µM	(88, 230, 262)
**PKC**		GF109203X	20 µM	(230)
		Phloretin	25 µM	(262)
		BIM	2 µM	(268)
**PI3K**		Wortmannin	10–100 nM	(146, 164)
		LY294002	10 µM	(164)
**JAK2**		AG490	100 µM	(146)
**MEK1/2**		PD98059, U0126	10 µM	(62, 134, 164)
**p38 MAPK**		SB202190	20 µM	(146)
		SB203580, PD169816	10 µM	(164)
**Transcription**		Actinomycin D	8 µM	(129)

## Perspectives

While GH is a key endocrine regulator of somatic growth, it is also involved in the regulation of other vital processes in vertebrates. Thus, GH has implications in health, disease and even in animal production, and the fine–tuned control of GH synthesis and secretion is still a hot research topic more than 75 years after its discovery (1). Numerous GH regulators have been discovered and more progress in our knowledge on GH and somatotroph biology is expected in the future. Definitively, the progress in our knowledge of GH and its transfer and application will benefit the society in many ways. The same reasons support the need for more basic, clinical and comparative endocrinology research on GH biology in vertebrates.

## Author Contributions

EV and SU prepared the manuscript, and EV created the tables and figures. All authors contributed to the article and approved the submitted version.

## Funding

EV is supported by postdoctoral fellowship (#4530) and a Top–Up Incentive Award (#5362) from the Saskatchewan Health Research Foundation (SHRF), and a postdoctoral fellowship (#2019MFE–429976–71377) from the Canadian Institutes of Health Research (CIHR). SU is a University of Saskatchewan Centennial Enhancement Chair in Comparative Endocrinology, and the research on hormones in his laboratory has been funded by grants from the Natural Sciences and Engineering Research Council (NSERC), CIHR, Canada Foundation for Innovation, SHRF, and Ferring Pharmaceuticals.

## Conflict of Interest

The authors declare that the research was conducted in the absence of any commercial or financial relationships that could be construed as a potential conflict of interest.
